# Proteomic and Metabolomic Analyses of HPV-Positive High-Grade Squamous Intraepithelial Lesions

**DOI:** 10.3390/biomedicines14040745

**Published:** 2026-03-24

**Authors:** Chengcheng Zhao, Yan Li, Yingfei Lu, Tianming Wang, Jianquan Chen

**Affiliations:** 1 Central Laboratory, Nanjing Jiangning Hospital, Nanjing 211100, China; zhao_chch@163.com (C.Z.); emma_0614@163.com (Y.L.); 2 Clinic Laboratory, Nanjing Jiangning Hospital, Nanjing 211100, China; jnyymys@163.com

**Keywords:** proteomics, metabolomics, integrative pathway analysis, CPT1A, high-grade squamous intraepithelial lesion

## Abstract

**Background/Objectives**: Long-term exposure to high-risk human papilloma virus (HPV) leads to high-grade squamous intraepithelial lesions (HSILs), which may develop into cancer. Various proteins and metabolites change during the development of cervical cancer; thus, assessing the dysregulated molecules and pathways in HSILs is important to elucidate early pathological mechanisms and identify potential intervention targets. **Methods**: In this study, we performed proteomic and metabolomic analyses in five pairs of HPV-positive HSIL tissues and paired normal tissues. Immunohistochemistry (IHC) was applied to validate the levels of carnitine palmitoyltransferase 1A (CPT1A) in HSIL tissues. Quantitative real-time PCR and Western blot were used to detect the expression levels of CPT1A in cervical cancer cell lines. **Results**: In proteomic analysis, 836 proteins showed significant changes. Functional analyses of the differentially expressed proteins indicated that metabolic pathways, oxidative phosphorylation and ribosome are the top three enriched pathways. In metabolomic analysis, 105 metabolites were differentially altered. Most metabolites were involved in lipid metabolism, such as phosphatidylethanolamine (PE), phosphatidylinositol (PI) and L-palmitoylcarnitine. Integrated proteomics and metabolomics revealed that the metabolic pathway was the most enriched pathway that contained the maximum number of differentially expressed metabolites and proteins. In vitro, we found CPT1A was upregulated in HSIL tissues and in cervical cancer cell lines. **Conclusions**: Our findings characterize the protein and metabolite alterations in HSILs, which may represent molecular features associated with disease progression.

## 1. Introduction

According to the global cancer statistics from the World Health Organization, cervical cancer is the fourth most prevalent cancer and the third leading cause of cancer death in women worldwide [1]. HPV infection is the major causative factor of cervical cancer [2]. In reproductive-age women, HPV infections are very common. In most cases, infections of HPV can be cleared up spontaneously by the host immune system. However, long-term exposure to high-risk HPV subtypes such as HPV16 and HPV18 might proceed to a cervical squamous intraepithelial lesion (SIL) and ultimately cancer [3].

According to the cervical lesion grade, SILs can be classified into low-grade SILs (LSILs) and high-grade SILs (HSILs) [4]. It is recommended that LSILs be used as a diagnostic category to describe lesions associated with transient HPV infection [5]. HSIL is a precancerous lesion of the cervix. Compared with an LSIL or normal tissue, an HSIL exhibits obvious malignant biological behaviors [6]. Without treatment, about one-third of HSILs may progress to invasive cervical cancer [7]. The preferred treatment for HSILs is cervical conization, which might impair the fertility of patients. In addition, a proportion of HSIL cases may recur or remain infected with HPV after treatment. Therefore, studying HSILs may help to identify early molecular events underlying malignant progression.

Previous studies have reported that multiple proteins and metabolites are altered in serum and tissue samples of HSIL patients [8,9,10,11,12,13]. Data-independent acquisition analysis combined with ELISA was used to screen and validate two potential biomarkers for early diagnosis of HSILs [12]. Xu et al. reported that microbiomic and metabolomic profiling can distinguish cervical cancer from a precancer lesion and identify potential biomarkers for the early detection of cervical dysplasia [8]. However, these studies have been limited to either proteomic or metabolomic analysis. Proteomic profiles reflect the functional changes at the protein level, while metabolic profiles represent the final phenotypic output of biological activity. Therefore, integrative pathway analysis of proteomics and metabolomics could provide a more complete understanding of the biological mechanisms of HSILs. In this study, we performed proteomic and metabolomic analyses in HPV-positive HSIL tissues to identify dysregulated molecules and pathways associated with disease progression.

## 2. Materials and Methods

### 2.1. Tissue Samples

In the proteomic and metabolomic analyses, the five pairs of HPV-positive HSIL tissue and paired normal tissue used were obtained from residual specimens after testing in Nanjing Jiangning Hospital. All patients were diagnosed with HSILs for the first time. Patients with other gynecological diseases or tumors were excluded. Tissue samples were stored in liquid nitrogen until use after collection. The clinical characteristics of the patients are listed in Table 1.

### 2.2. Label-Free Quantitative Proteomic Analysis

Proteomic analysis was conducted by the Shanghai Bioprofile Technology Company (Shanghai, China). Proteins were extracted from tissue samples using SDT lysis buffer. The lysed samples were boiled for 3 min and sonicated for 2 min. After centrifugation at 16,000× *g* for 20 min, the supernatant was collected for protein quantification. Protein concentration was determined using the BCA assay. Briefly, 200 μg of protein from each sample was digested by using the filter-aided sample preparation (FASP) method to collect peptides as previously described [14], and the resulting peptides were collected and quantified. All samples were processed in parallel using the same batch of reagents to avoid batch effects. Equal amounts of peptides from each sample were subjected to LC-MS analysis on a Q-Exactive Plus mass spectrometer coupled with an Easy-nLC 1200 system (Thermo Fisher Scientific, Bremen, Germany). Peptides were loaded onto a C18 trap column (100 μm × 20 mm, 5 μm, Dr. Maisch GmbH, Ammerbuch, Germany) equilibrated with buffer A (0.1% formic acid in water). Reverse-phase high-performance liquid chromatography (RP-HPLC) separation was performed using a self-packed column (75 μm × 150 mm; 3 μm ReproSil-Pur C18 beads, 120 Å, Dr. Maisch GmbH, Ammerbuch, Germany) at 300 nL/min. The RP-HPLC mobile phase A consisted of 0.1% formic acid in water, and mobile phase B consisted of 0.1% formic acid in 95% acetonitrile. Peptides were eluted using a 120 min linear gradient. MS analysis was operated in data-dependent top20 mode (300–1800 *m*/*z*) with higher-energy collisional dissociation (HCD) fragmentation. A lock mass of 445.120025 Da was used for internal calibration. Full MS scans were acquired at a resolution of 70,000 (at *m*/*z* 200) and MS/MS scans at 17,500 (at *m*/*z* 200), with a maximum injection time of 50 ms for both. The normalized collision energy was 27%, with an isolation window of 1.6 Th and dynamic exclusion of 60 s. LC-MS/MS data were analyzed using the Proteome Discoverer software (version 2.4) and searched against the UniProtKB *Homo sapiens* (Human) database (downloaded 17 July 2023, 207,981 total entries, containing both Swiss-Prot and TrEMBL entries). The parameters were set as follows: up to two missed cleavage sites; precursor ion mass tolerance, 4.5 ppm; fragment ion mass tolerance, 20 ppm. Carbamidomethylation of cysteine was defined as a fixed modification, while N-terminal acetylation and methionine oxidation were set as variable modifications. These are routine modifications for standard peptide identification and were not used for dedicated identification of post-translational modifications. The results were filtered with a false discovery rate (FDR) < 1% at both peptide-spectrum match and protein levels. Label-free quantification (LFQ) was performed in MaxQuant using established intensity-based algorithms as previously described [15,16,17]. Protein intensities were calculated at the protein group level based on pairwise peptide comparisons. For gene-level functional enrichment analysis, protein groups mapping to the same gene were collapsed by retaining the entry with the highest LFQ intensity as the representative quantitative value for that gene. This step was performed after quantification to ensure each gene was represented by its most abundant product, thereby avoiding statistical redundancy in subsequent enrichment analyses. Proteins with a fold change (FC) ≥ 2 or FC < 0.5 and *p* < 0.05 were considered significantly differentially expressed.

### 2.3. Metabolomic Analysis

Untargeted metabolomics was performed by the Shanghai Bioprofile Technology Company (Shanghai, China) as previously described using the same tissues as for the proteomic analysis [18]. After weighing, tissue samples were homogenized with a precooled mixture of methanol and water at a volume ratio of 4:1. The mixture was sonicated in an ice bath for 20 min, followed by incubation at −20 °C for 1 h. After centrifugation at 16,000× *g* for 20 min at 4 °C, the supernatants were collected and dried under vacuum. Metabolomic profiling was performed using a Shimadzu Nexera X2 LC-30AD UHPLC system coupled with Q-Exactive Plus (Thermo Scientific, San Jose, CA, USA). Chromatographic separation was carried out on an ACQUITY UPLC HSS T3 column (2.1 × 100 mm, 1.8 μm; Waters, Milford, MA, USA) at 0.3 mL/min. Mobile phase A was 0.1% formic acid in water, and mobile phase B was 100% acetonitrile. The gradient elution was programmed as follows: 0% B for 2 min, linearly increased to 48% B in 6 min, raised to 100% B in 10 min and held for 12 min, then returned to 0% B for re-equilibration. Electrospray ionization (ESI) was applied for MS data acquisition in both positive and negative modes. The HESI source parameters were as follows: Spray Voltage: 3.8 kV (positive)/3.2 kV (negative); Capillary Temperature: 320 °C; Sheath Gas (nitrogen) flow: 30 arb (arbitrary units); Aux Gas flow: 5 arb; Probe Heater Temperature: 350 °C; S-Lens RF Level: 50. Full MS scans were recorded over 70–1050 Da at 70,000 resolution and MS/MS scans at 17,500 resolution. The maximum injection times were 100 ms for MS and 50 ms for MS/MS. The isolation window for MS2 was set to 2 *m*/*z* and the normalized collision energy (stepped) was set as 20, 30 and 40 for fragmentation. Raw MS data were processed using MS-DIAL (v4.90, RIKEN, Saitama, Japan) for peak alignment, retention time correction and peak area extraction. The metabolites were identified by accuracy mass (mass tolerance < 10 ppm) and MS/MS data (mass tolerance < 0.02 Da), which were matched with HMDB, Massbank and other public databases and our self-built metabolite standard library.

### 2.4. Immunohistochemistry (IHC)

The paraffin blocks of the HSIL and control (endocervicitis or normal tissues) used in IHC were from the pathology department of Nanjing Jiangning Hospital. Paraffin sections underwent dewaxing and rehydration before antigen retrieval. Sections were treated with H_2_O_2_ to block endogenous substances and then incubated with 5% BSA to reduce non-specific binding sites. The sections were incubated with CPT1A rabbit polyclonal antibody (1:100, A5307, RRID: AB_2766119, Abclonal, Wuhan, China) overnight at 4 °C, followed by incubation with the secondary antibody, and finally subjected to DAB staining. Images were captured by a microscope (Nikon, Tokyo, Japan), and the expression levels of CPT1A were calculated by Image J software (v1.53t, National Institutes of Health, Bethesda, MD, USA).

### 2.5. Cell Culture

Cervical cancer cell lines were purchased from Haixing Biosciences (Suzhou, China). Cells were grown in Dulbecco’s modified Eagle medium (BC-M-005, Biochannel, Nanjing, China) with 10% FBS (F103-01, Vazyme, Nanjing, China), 1% penicillin and streptomycin (BC-CE-007, Biochannel). The culture condition of the cells was 37 °C with 5% CO_2_.

### 2.6. Quantitative Real-Time PCR (qRT-PCR)

RNA was extracted from cervical cancer cells (RC101-01, Vazyme) following the instructions of the manufacture. cDNA was synthesized using HiScript III All-in-one RT SuperMix Perfect for qPCR (R333-01, Vazyme) to remove genomic DNA. PCR was carried out using ChamQ SYBR qPCR Master Mix (Q341-02, Vazyme) on ABI QuantStudio5 (Applied Biosystems, Foster City, CA, USA). The PCR reaction conditions were 95 °C for 30 s followed by 40 cycles on 95 °C for 10 s and 60 °C for 30 s following the manufacturer’s protocol. Primer sequences were as follows: CPT1A forward: ATCAATCGGACTCTGGAAACGG, CPT1A reverse: TCAGGGAGTAGCGCATGGT, β-Actin forward: AAAGACCTGTACGCCAACAC, β-Actin reverse: GTCATACTCCTGCTTGCTGAT. The relative mRNA expression level of CPT1A was normalized using β-Actin expression as an internal reference and analyzed by using the 2^−δδCT^ method.

### 2.7. Western Blot Assay

Cervical cancer cells were washed with cold PBS twice and then lysed using RIPA lysis buffer (P0013, Beyotime, Nantong, China). After centrifugation at 13,000× *g* for 15 min, the supernatant containing protein was collected and quantified by using the BCA kit (P0012S, Beyotime, China). Next, 20 μg protein of each sample was separated by electrophoresis on a SurePAGE™, Bis-Tris, 10% gel (M00664, Genscript, Nanjing, China) at 120 V for 1 h. Proteins and protein markers (WJ103, Epizyme Biotech, Shanghai, China) on the gel were transferred onto a methanol-activated PVDF membrane (88518, Milipore, Darmstadt, Germany) in a precooled transfer buffer (M00139, Genscript) at 250 mA for 1 h. The membrane was blocked with QuickBlock Blocking Buffer (P0252, Beyotime) at room temperature for 20 min and then incubated with CPT1A rabbit polyclonal antibody (1:1000, A5307, Abclonal) and rabbit monoclonal β-Actin (1:1000, AC026, Abclonal) at 4 °C overnight. After incubation with secondary antibody (1:5000, AS014, Abclonal) at room temperature for 1 h, the membrane was visualized by BeyoECL Plus (P0018S, Beyotime) at FluorChem E (ProteinSimple, San Jose, CA, USA). Protein band intensities were quantified using ImageJ software. The relative expression levels of CPT1A were normalized to β-Actin. The experiments were performed in biological triplicate.

### 2.8. Statistical Analysis

Bioinformatic analyses of the proteomic and metabolomic data were performed using Perseus software (v1.6.15.0, Max Planck Institute of Biochemistry, Martinsried, Germany), Microsoft Excel and R (v4.0.3, R Foundation for Statistical Computing, Vienna, Austria) statistical computing software. Hierarchical clustering analysis was performed using the pheatmap package in R. Gene Ontology (GO) and Kyoto Encyclopedia of Genes and Genomes (KEGG) enrichment analyses were carried out using Fisher’s exact test, with enriched GO terms and KEGG pathways considered statistically significant at the *p* < 0.05 level. Integrative pathway analysis of proteomics and metabolomics were performed using R, and the resulting interaction networks were visualized with Cytoscape software (v3.9.1, Cytoscape Consortium, Seattle, WA, USA). Differences between two groups were analyzed using Student’s *t*-test, and comparisons among multiple groups were performed using one-way analysis of variance (ANOVA). *p* < 0.05 was regarded as significantly difference.

## 3. Results

### 3.1. Altered Proteomic Profiles in HSIL Tissues

**Figure 1:**
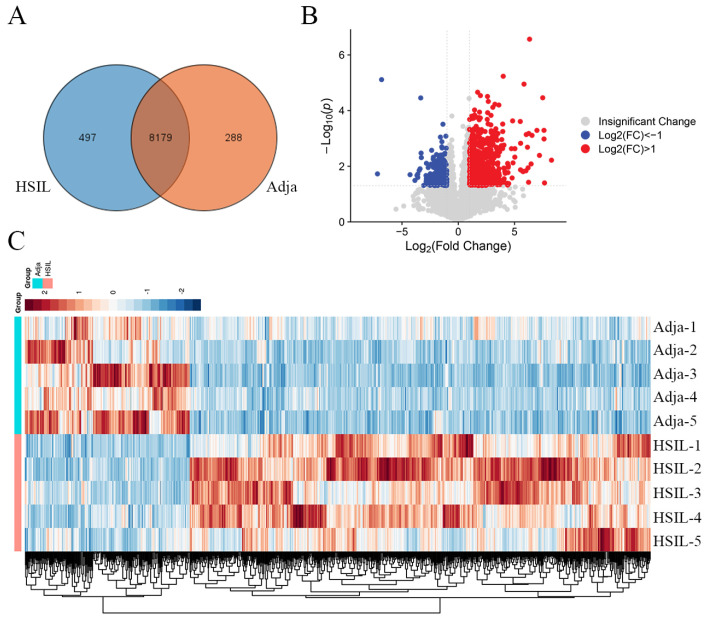
Altered proteomic profiles in HSIL tissues compared with adjacent normal tissue. (**A**) Number of proteins detected in HSIL tissues and paired adjacent normal tissues. (**B**) Volcano plot showing significantly differentially expressed proteins between HSIL and adjacent tissues. (**C**) Heatmap of the significantly differentially expressed proteins between the two groups. HSIL: high-grade squamous intraepithelial lesion. Adja: adjacent normal tissue. FC: fold change.

We performed proteomic analysis on five pairs of HPV-positive HSIL and adjacent tissue samples to detect differences in protein expression between the two groups. In total, 58,454 peptides and 8964 proteins were identified. Among the 8964 proteins, 8179 were shared in the HSIL and control groups (Figure 1A). The expression levels of 836 proteins were significantly altered (FC > 2.0 or FC < 0.5 and *p* < 0.05). Of these, 616 proteins were significantly increased, and 220 proteins were significantly decreased in HSIL tissues when compared with the adjacent normal tissues (Figure 1B). The heatmap revealed that HSIL tissues had a different proteomic pattern from adjacent normal tissues (Figure 1C).

### 3.2. GO and KEGG Enrichment Analyses of the Differentially Expressed Proteins

**Figure 2:**
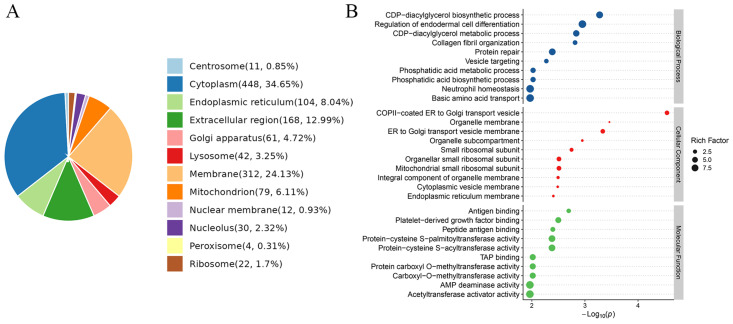
Functional annotation of the significantly differentially expressed proteins between HSIL tissues and paired adjacent normal tissues. (**A**) Subcellular localization of the differentially expressed proteins. Numbers in parentheses indicate the count and percentage of proteins distributed to each subcellular component. (**B**) The top 10 significantly enriched GO terms (*p* < 0.05), including biological process, molecular function, and cellular component. GO: Gene Ontology.

The subcellular localization of protein is crucial in understanding protein structure, function and protein–protein interactions, so we analyzed the subcellular localization of the differentially expressed proteins. The results showed that the top three subcellular localization regions were cytoplasm, membrane and extracellular region (Figure 2A). The GO database was used to classify the identified proteins into biological process (BP), molecular function (MF) and cellular component (CC) terms. The top 10 terms with significant enrichment of differentially expressed proteins in the BP, MF, and CC branches are displayed in Figure 2B. The BP enriched terms showed that the enriched proteins were related to cytidine diphosphate diacylglycerol (CDP-DAG), such as CDP-DAG biosynthetic process and CDP-DAG metabolic process. Furthermore, regulation of endodermal cell differentiation, collagen fibril organization and protein repair were also enriched. The CC enriched terms showed that COPII-coated ER to Golgi transport vesicle, organelle membrane, and ER to Golgi transport vesicle membrane were highly enriched. The MF enriched terms showed that differentially expressed proteins were involved in enzyme activity, such as protein–cysteine S-palmitoyltransferase activity, protein–cysteine S-acyltransferase activity, protein carboxyl-O-methyltransferase activity, carboxyl-O-methyltransferase activity, AMP deaminase activity and acetyltransferase activator activity. Antigen binding, platelet-derived growth factor binding, peptide antigen binding and TAP binding were also enriched.

**Figure 3:**
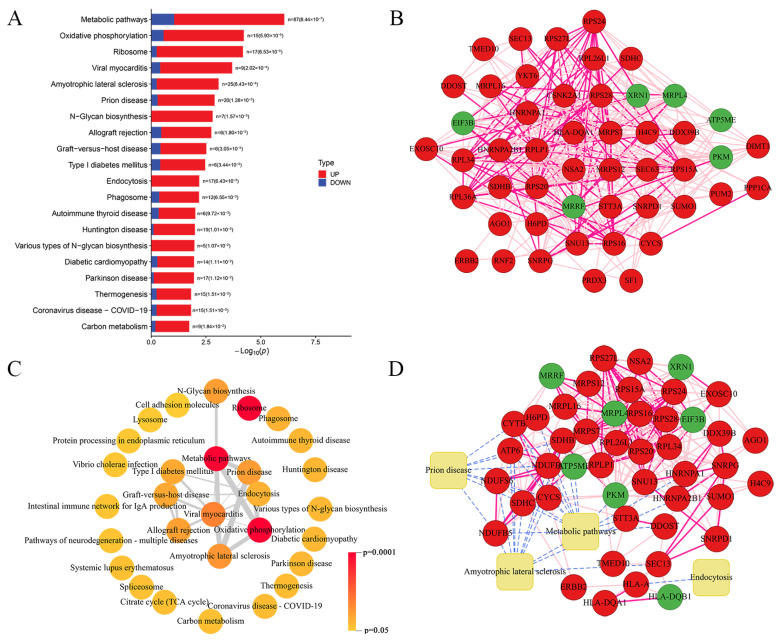
KEGG enrichment analysis and protein interaction network of the differentially expressed proteins between HSIL tissues and paired adjacent normal tissues. (**A**) The top 20 significantly enriched KEGG pathways of the differentially expressed proteins (*p* < 0.05). (**B**) Protein–protein interaction network constructed from the differentially expressed proteins. Red indicates upregulation and green indicates downregulation. (**C**) Pathway–pathway relationship network based on the enriched KEGG pathways. The thicker the line, the more elements they share. (**D**) Protein–protein interaction network of the top 50 proteins ranked by PageRank, with corresponding significantly enriched pathway information annotated. Red circles represent upregulated proteins, green circles represent downregulated proteins, and yellow boxes represent metabolic pathways.

Pathway enrichment analysis was conducted using the KEGG database. The top 20 enriched KEGG pathways are shown in Figure 3A. Oxidative phosphorylation, ribosome, and viral myocarditis were significantly enriched. Studying the protein–protein interactions is crucial for elucidating protein functions, so we integrated protein–protein interactions with pathway–protein relationships from the STRING database to construct the protein–protein interaction network (Figure 3B) and the pathway–pathway relationship network (Figure 3C). Moreover, we employed the PageRank algorithm to select the top 50 vertices, constructing a protein–protein interaction network diagram containing information on enriched pathways (Figure 3D).

### 3.3. Altered Metabolomic Profiles in HSIL Tissues

**Figure 4:**
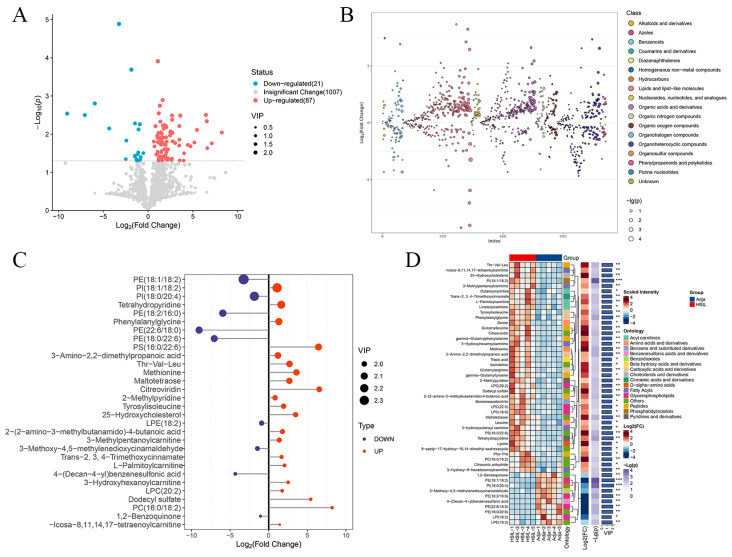
Altered metabolomic profiles in HSIL tissues compared with paired adjacent normal tissues. (**A**) The volcano plot displaying the differential metabolites detected in HSIL tissues and paired adjacent normal tissues (VIP > 1 and *p* < 0.05). Values in parentheses represent the count of detected metabolites per group. (**B**) Bubble plot classifying differential metabolites by FC and *p*-values in HSIL tissues compared with paired adjacent normal tissues. (**C**) Top 30 metabolites ranked by VIP values and fold change. (**D**) Hierarchical heatmap of the top 50 metabolites screened by VIP values. VIP: variable importance in projection. * *p* < 0.05, ** *p* < 0.01, *** *p* < 0.001.

In the Partial Least Squares Discriminant Analysis (PLS-DA) model, the R2 and Q2 values were 0.998 and 0.689. In the Orthogonal Partial Least Squares Discriminant Analysis (OPLS-DA) model, the R2 and Q2 values were 0.998 and 0.485. The Q2 of 200 permutation tests was −0.12, which suggests the original model is not overfitted. Metabolites showing significant differences were identified using the criteria of variable importance in projection (VIP) > 1 and *p* < 0.05. Overall, 105 significantly differentially expressed metabolites were identified between the HSIL and control groups, with 87 metabolites enhanced and 20 metabolites deficient (Figure 4A). The bubble chart displays the distribution of the metabolites between the two groups (Figure 4B). The top 30 important metabolites according to VIP are displayed in Figure 4C. Most metabolites were involved in lipid metabolism, such as phosphatidylethanolamine (PE), phosphatidylinositol (PI), and L-palmitoylcarnitine. The hierarchical clustering map shows the differential metabolites (top 50 VIP values) between the two groups (Figure 4D).

### 3.4. Differential Metabolites’ Functional Annotation

**Figure 5:**
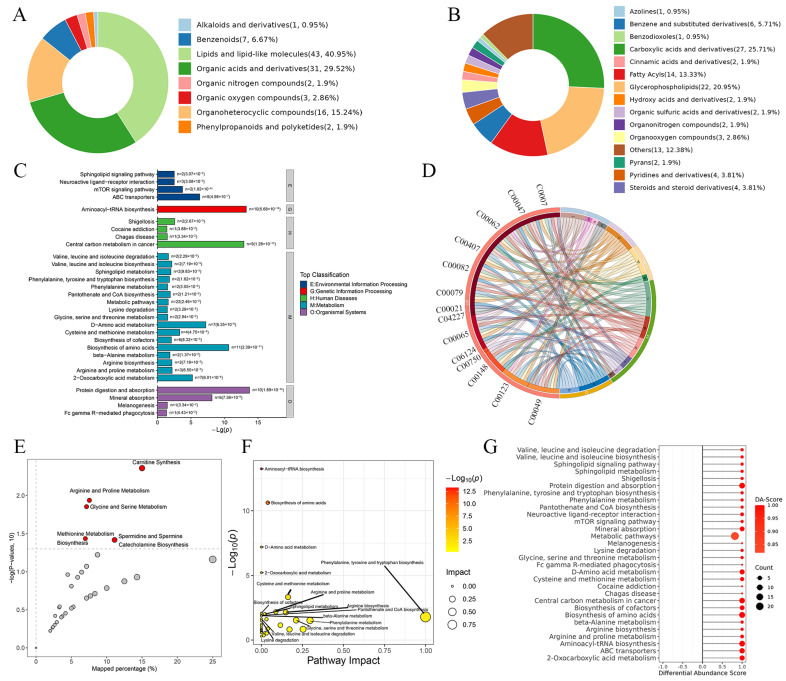
Functional annotation of differential metabolites between HSIL tissues and paired adjacent nor-mal tissues. The superclass (**A**) and class (**B**) classification of differential metabolites. Numbers in parentheses indicate the metabolite count per superclass/class. (**C**) The top 30 significantly enriched KEGG pathways based on differential metabolites. (**D**) Chord plot displaying the association of dysregulated metabolites with KEGG pathways between HSIL and Adja groups. (**E**) The significantly enriched metabolic pathways in the SMPDB. (**F**) The pathway impact map integrating metabolite interaction network and pathway enrichment analysis results. (**G**) Differential abundance (DA) scores of metabolites involved in the top 30 pathways. SMPDB: Small Molecule Pathway Database.

The differentially expressed metabolites in the two groups were classified according to their structure and function in the Human Metabolome Database (HMDB). Figure 5A,B display the superclass and class of the differentially expressed metabolites. The top three super classes of the differential metabolites were lipids and lipid-like molecules, organic acids and derivatives, and organoheterocyclic compounds. The top three classes of the differential metabolites were carboxylic acids and derivatives, glycerophospholipids and fatty acyls. KEGG enrichment analyses showed that the ABC transporter in environmental information processing, aminoacyl-tRNA biosynthesis in genetic information processing, central carbon metabolism in cancer in human diseases, biosynthesis of amino acids in metabolism, protein digestion and absorption in organismal systems showed the highest enrichment level (Figure 5C). The string diagram displays the top 15 enriched pathways and their associated differentially expressed metabolites (Figure 5D). The bubble chart shows pathways significantly enriched in the Small Molecule Pathway Database (SMPDB) (Figure 5E). Carnitine synthesis, arginine and proline metabolism, glycine and serine metabolism, methionine metabolism, catecholamine biosynthesis, spermidine and spermine biosynthesis were enriched. The network of the top 30 metabolites identified through pathway impact analysis is shown in Figure 5F. To systematically investigate the metabolic changes in the HSIL group, we analyzed the overall trend of the metabolic pathways based on the abundance of different metabolites. The differential abundance scores (DA score) indicated the overall trend of increased/decreased metabolites in the pathway relative to the control group. The data indicated that the metabolites in the top 30 enriched pathways all displayed increased trends (Figure 5G).

### 3.5. Integrative Pathway Analysis of Metabolomics and Proteomics

**Figure 6:**
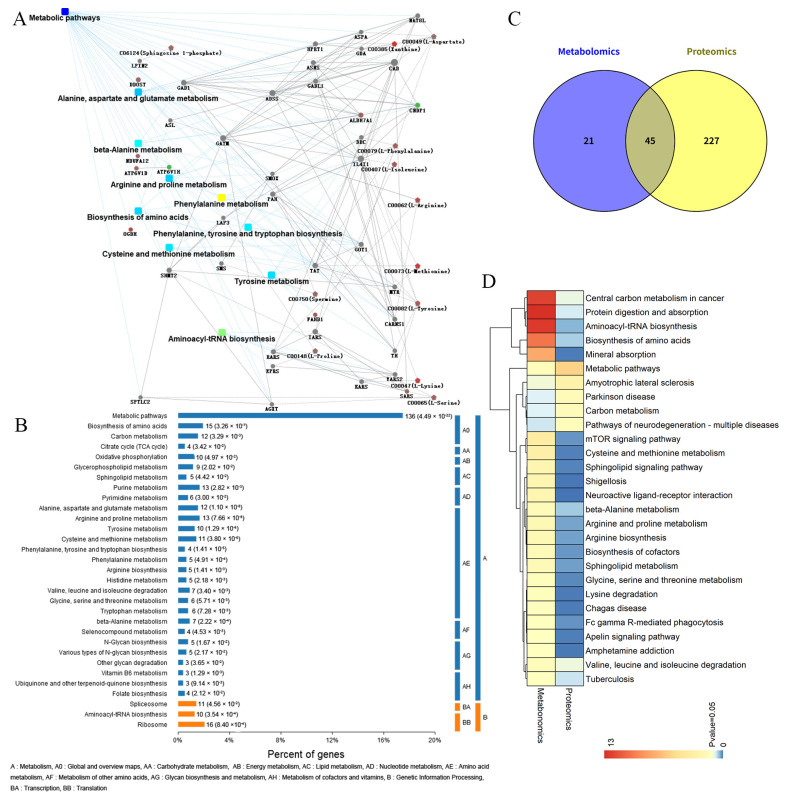
Integrative pathway analysis of metabolomics and proteomics between HSIL tissues and paired adjacent normal tissues. (**A**) Network constructed by integrating differential metabolomic and proteomic data. Rectangular nodes represent KEGG pathways, with a yellow-to-blue gradient indicating significance (*p*-value): yellow represents smaller *p*-values and blue represents larger *p*-values. Circular nodes denote proteins/genes, and pentagonal nodes represent metabolites, red indicates upregulation and green indicates downregulation. (**B**) The plot shows the enriched KEGG pathways from integrated proteomic and metabolomic data. Numbers in parentheses represent the *p* value. (**C**) The number of shared pathways between metabonomic and proteomic analyses. (**D**) The heatmap of shared KEGG pathways between metabonomic and proteomic analyses.

To further explore the association between differential proteins and metabolites, we conducted a combined analysis. The network between differentially expressed proteins and metabolites was analyzed, and the results are displayed in Figure 6A. Results from KEGG pathway analysis revealed that metabolic pathways were the most enriched pathway, which contained the largest number of differentially expressed metabolites and proteins. Within the metabolism category, amino acid metabolism, lipid metabolism, and energy metabolism were significantly enriched (Figure 6B). In total, 45 KEGG pathways were shared between differentially expressed metabolites and proteins (Figure 6C). The most enriched shared KEGG pathways related to amino acid metabolism and protein biosynthesis and central carbon metabolism in cancer (Figure 6D).

### 3.6. Protein Validation

**Figure 7:**
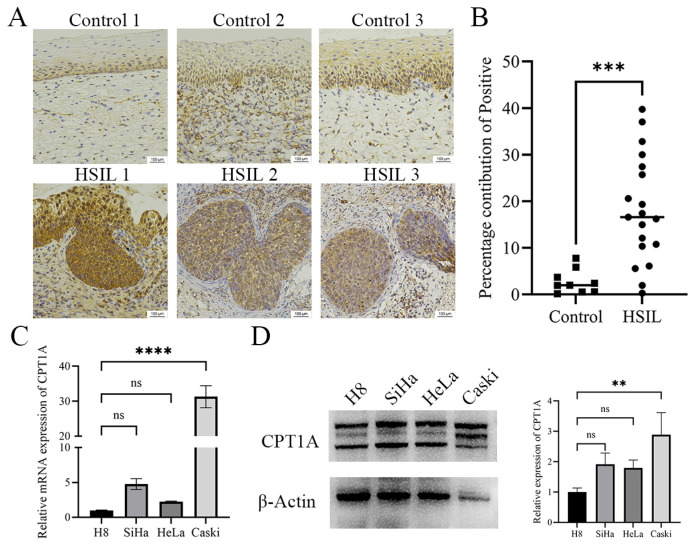
The expression levels of CPT1A in HSIL tissues and cervical cancer cell lines. (**A**) Representative IHC images show the protein expression levels of CPT1A in 3 HSIL tissues and 3 control tissues. (**B**) Scatter plot displays the expression levels of CPT1A in 19 HSIL tissues and 9 control tissues. (**C**,**D**) The mRNA and protein (upper band) expression levels of CPT1A in cervical cancer cell lines examined by qRT-PCR and Western blot assay. ** *p* < 0.01, *** *p* < 0.001, **** *p* < 0.0001, ns (not significant).

The metabolomic data showed that L-palmitoylcarnitine was enriched. CPT1A catalyzes the formation of L-palmitoylcarnitine. We next validated the expression levels of CPT1A in HSIL tissues and cell lines. IHC was performed in 19 cases of HPV-positive HSIL tissues and 9 cases of HPV-positive control tissues. The characteristics of the subjects are listed in Table 2. The results showed that CPT1A was significantly upregulated in HSIL tissues as compared with control tissues (Figure 7A,B). We further verified the expression pattern of CPT1A in three cervical cancer cell lines (SiHa, HeLa and Caski) and one immortalized normal cervical epithelial cell line (H8). The results showed that CPT1A was elevated in both mRNA (Figure 7C) and protein (Figure 7D) levels in the three cervical cancer cell lines (SiHa, HeLa and Caski) as compared with H8, especially in the Caski cell line.

## 4. Discussion

At present, studies on proteomic and metabolic changes in cervical cancer have been largely reported [19,20,21], but studies on such changes in HSILs remain limited. Dimitra Pouli et al. found that metabolic alterations were detected in human SIL tissues [9]. In this study, we found 616 upregulated and 220 downregulated proteins in HSIL tissues as compared with the adjacent tissues. Meanwhile, 105 metabolites between HSIL and control groups showed differential levels [18]. Integrative pathway analysis of proteomics and metabolomics showed that metabolic pathway was the most enriched pathway.

By using the function analysis of the differential expressed proteins in this study, we found CDP-DAG biosynthetic process and CDP-DAG metabolic process were significantly enriched. CDP-DAG is a key intermediate in lipid metabolism, which is a precursor of phosphatidylinositol (PI) and cardiolipin (CL) [22]. PI and CL play important roles in signal transduction, membrane traffic, and maintenance of the mitochondrial function [22]. The alterations in CDP-DAG biosynthetic and metabolic processes indicated the deregulation of lipid metabolism in HSILs. In carcinogenesis, lipid metabolism is reprogrammed for membrane biogenesis and energy storage [23]. Yong Chen et al. found that HSILs displayed specific lipid accumulation. Mainly, the levels of triglycerides and phosphatidylcholines were enhanced, while the levels of lipids were deficient [24]. Katarzyna Sitarz et al. reported that the lipid level is significantly elevated in the cytoplasm of cervical epithelial cells in the HSIL [25]. Our results suggest CDP-DAG might be an essential target for regulating lipid homeostasis of HSILs. In KEGG analysis, we found metabolic pathways were the most significantly enriched pathway. Metabolic pathways include glycolysis, fatty acid oxidation, and amino acid metabolism, which play vital roles in cellular homeostasis. During carcinogenesis, various cellular energy metabolic pathways were altered to facilitate tumor cell proliferation and modify the microenvironment [26]. The most well-known metabolic change in cancer is the enhanced glycolysis [27]. In cervical cancer, cells tend to obtain energy through glycolysis rather than oxidative phosphorylation [28]. In HSIL tissues, we found that oxidative phosphorylation was more enhanced than that in control tissues. Our results were consistent with another research that reported that fatty acid metabolism and oxidative phosphorylation were upregulated in HSIL tissues [11]. Thus, we speculate that the glucose metabolism patterns are different in HSILs and cervical cancer.

In metabonomic analysis, we found most of the top 30 important metabolites were involved in lipid metabolism, such as phosphatidylethanolamine (PE), phosphatidylinositol (PI) and L-palmitoylcarnitine. L-Palmitoylcarnitine is an important endogenous metabolite in the β-oxidation of fatty acids, which is synthesized from palmitoyl-CoA and L-carnitine by CPT1A [29]. The metabonomic analysis showed that L-palmitoylcarnitine was enhanced in HSIL tissues. Moreover, the HMDB analysis identified that the carnitine synthesis was significantly enriched. There was no report on the role of L-palmitoylcarnitine in HSILs and cervical cancer. Feng Qiu et al. reported that L-carnitine was continuously upregulated in serum of healthy people and LSIL and HSIL groups [10]. These results further suggested that fatty acid oxidation was altered in HSILs. Given the exploratory nature and relatively small sample size of the present study, FDR correction was not performed for the identification of differential metabolites, which may lead to an elevated risk of false-positive findings. Therefore, future validation in large independent cohorts is warranted to verify the identified metabolic alterations. In addition, we did not verify the levels of L-palmitoylcarnitine in HSIL tissues in the present study. The concentration of L-palmitoylcarnitine will be further validated using targeted metabolomic analysis.

CPT1A is located in the outer membrane of mitochondria, which is a rate-limiting enzyme in fatty acid β-oxidation [30]. CPT1A is a key driver of tumor progression, participating in multiple stages of cancer development and metastasis [31,32]. Previous studies have reported that CPT1A facilitated the metastasis of cervical cancer cells via lipid metabolism [33,34]. To the best of our knowledge, no relevant studies have reported the expression profile and function of CPT1A in HSILs. The IHC staining results in the present study verified that the HSIL tissues displayed a higher expression level of CPT1A than the control tissues. Further validation in a larger sample size will be conducted to evaluate the diagnostic value of CPT1A in future research. Since there was no HSIL cell line, the expression of CPT1A was evaluated in cervical cancer cell lines. We found both the mRNA and protein levels of CPT1A were significantly higher in three cervical cancer cell lines than in H8 cells. These results suggest that changes in the expression of CPT1A might play an important role in HSILs and cervical cancer.

Through mapping proteomic and metabolomic data onto KEGG pathways, we found 45 overlapping KEGG pathways, and metabolic pathways were the most significantly enriched. Pathways related to amino acid metabolism and protein biosynthesis as well as central carbon metabolism in cancer were significantly enriched in both proteomics and metabolomics, indicating a coordinated dysregulation of metabolic and translational processes. The combined analysis of proteomics and metabolomics in the present study is primarily based on biological interpretation; future studies will focus on integrated proteomic and metabolomic modeling, correlation analysis of protein–metabolite pairs, and regulatory network construction.

A major limitation of this study was the relatively small sample size in proteomic and metabolomic analysis, which may be underpowered for robust validation of the identified candidate molecules. Although we identified differentially expressed proteins and metabolites, the findings should be further verified in large-scale independent cohorts. In addition, the present study validated the expression levels of CPT1A in HSIL tissues and cervical cancer cell lines. Functional studies in HSIL-relevant models, investigation of the relationship between CPT1A upregulation and HSIL progression, and investigations into the relationship between CPT1A expression and HPV oncoprotein biology were not performed. Future studies will focus on functional validation and mechanistic exploration of the CPT1A/L–palmitoylcarnitine axis in HSILs. The relationship between CPT1A and HSIL progression will be explored in HSIL animal models. In addition, the correlation between CPT1A expression and HPV status, viral load, or E6/E7-mediated signaling pathways will also be investigated to clarify the mechanism linking viral oncogenesis and metabolic reprogramming.

In conclusion, this study characterizes proteomic and metabolomic profiling in HSIL tissues, identifying distinct alterations in multiple proteins, metabolites, and pathways. Specifically, we found the levels of L-palmitoylcarnitine and CPT1A were altered in HSIL tissues. In addition, CPT1A displayed increased expression pattern in both HSIL tissues and cervical cancer cells. The proteomic and metabolic profiling might provide insights into the development of HSILs and may help identify potential diagnostic targets.

## Figures and Tables

**Table 1:** Clinical characteristics of patients included in proteomic and metabolomic analyses.

Age		42.2 ± 11.64
HPV infection	Single	3
	Multiple	2
Risk	High	5
	Middle	0
	Low	0

High-risk HPV subtype: HPV16, 18, 31, 33, 35, 39, 45, 51, 52, 56, 58, 59, 68; middle-risk HPV subtype: HPV26, 53, 66, 73, 82; low-risk HPV subtype: HPV6, 11, 40, 42, 43, 44, 54, 61, 70, 72, 81, 89.

**Table 2:** Clinical characteristics of patients included in IHC analysis.

		Control Group (*n* = 9)	HSIL Group (*n* = 19)
Age		53.10 ± 14.24	48.66 ± 14.60
HPV infection	Single	6	13
	Multiple	3	6
Risk	High	6	18
	Middle	3	1
	Low	0	0

High-risk HPV subtype: HPV16, 18, 31, 33, 35, 39, 45, 51, 52, 56, 58, 59, 68; middle-risk HPV subtype: HPV26, 53, 66, 73, 82; low-risk HPV subtype: HPV6, 11, 40, 42, 43, 44, 54, 61, 70, 72, 81, 89.

## Data Availability

The proteomic data have been deposited in the ProteomeXchange Consortium (https://proteomecentral.proteomexchange.org, accessed on 27 February 2026) via the iProX partner repository with the dataset identifier PXD074985. The metabolomic data have been submitted to MetaboLights as MTBLS13948. The data supporting the reported results of this article are available from the corresponding author upon reasonable request.

## Abbreviations

The following abbreviations are used in this manuscript:

HPV	Human papilloma virus
SIL	Squamous intraepithelial lesion
HSIL	High-grade squamous intraepithelial lesion
CPT1A	Carnitine palmitoyltransferase 1A
PE	Phosphatidylethanolamine
PI	Phosphatidylinositol
KEGG	Kyoto Encyclopedia of Genes and Genomes
IHC	Immunohistochemistry

## References

[1] Bray F., Laversanne M., Sung H., Ferlay J., Siegel R.L., Soerjomataram I., Jemal A. (2024). Global cancer statistics 2022: GLOBOCAN estimates of incidence and mortality worldwide for 36 cancers in 185 countries. CA Cancer J. Clin..

[2] Voelker R.A. (2023). Cervical Cancer Screening. JAMA.

[3] Hernandez-Silva C.D., Ramirez de Arellano A., Pereira-Suarez A.L., Ramirez-Lopez I.G. (2024). HPV and Cervical Cancer: Molecular and Immunological Aspects, Epidemiology and Effect of Vaccination in Latin American Women. Viruses.

[4] Li Y., Wang W., Xu D., Liang H., Yu H., Zhou Y., Liang J., Sun H., Liu X., Xue M. (2024). PIWIL2/PDK1 Axis Promotes the Progression of Cervical Epithelial Lesions via Metabolic Reprogramming to Maintain Tumor-Initiating Cell Stemness. Adv. Sci..

[5] Alrajjal A., Pansare V., Choudhury M.S.R., Khan M.Y.A., Shidham V.B. (2021). Squamous intraepithelial lesions (SIL: LSIL, HSIL, ASCUS, ASC-H, LSIL-H) of Uterine Cervix and Bethesda System. Cytojournal.

[6] Wei Y., Niu J., Gu L., Hong Z., Bao Z., Qiu L. (2024). Effect of Clinicopathological Characteristics on the Outcomes of Topical 5-Aminolevulinic Acid Photodynamic Therapy in Patients with Cervical High-Grade Squamous Intraepithelial Lesions (HSIL/CIN2): A Retrospective Cohort Study. Biomedicines.

[7] Perkins R.B., Wentzensen N., Guido R.S., Schiffman M. (2023). Cervical Cancer Screening: A Review. JAMA.

[8] Xu H., Liu L., Xu F., Liu M., Song Y., Chen J., Zhan H., Zhang Y., Xu D., Chen Y. (2022). Microbiome-metabolome analysis reveals cervical lesion alterations. Acta Biochim. Biophys. Sin..

[9] Pouli D., Thieu H.T., Genega E.M., Baecher-Lind L., House M., Bond B., Roncari D.M., Evans M.L., Rius-Diaz F., Munger K. (2020). Label-free, High-Resolution Optical Metabolic Imaging of Human Cervical Precancers Reveals Potential for Intraepithelial Neoplasia Diagnosis. Cell Rep. Med..

[10] Qiu F., Su B., Li Z., Chen W., Cao L., Chen F., Liu D., He J., Lin N. (2019). New serum biomarker identification and analysis by mass spectrometry in cervical precancerous lesion and acute cervicitis in South China. Cancer Manag. Res..

[11] Polleys C.M., Singh P., Thieu H.T., Genega E.M., Jahanseir N., Zuckerman A.L., Diaz F.R., Patra A., Beheshti A., Georgakoudi I. (2024). Rapid, high-resolution, non-destructive assessments of metabolic and morphological homogeneity uniquely identify high-grade cervical precancerous lesions. bioRxiv.

[12] Han S., Zhang J., Sun Y., Liu L., Guo L., Zhao C., Zhang J., Qian Q., Cui B., Zhang Y. (2022). The Plasma DIA-Based Quantitative Proteomics Reveals the Pathogenic Pathways and New Biomarkers in Cervical Cancer and High Grade Squamous Intraepithelial Lesion. J. Clin. Med..

[13] Xu J., Wang M., Jia Y., Chen Y., Duan Y., Ni M., Wang Y., Wei J., Yu J. (2025). Study on Differential Metabolites Between Human Papillomavirus Infection and Cervical Cancer Based on Non-Targeted Metabolomics. Int. J. Women’s Health.

[14] Wiśniewski J.R., Zougman A., Nagaraj N., Mann M. (2009). Universal sample preparation method for proteome analysis. Nat. Methods.

[15] Schwanhäusser B., Busse D., Li N., Dittmar G., Schuchhardt J., Wolf J., Chen W., Selbach M. (2011). Global quantification of mammalian gene expression control. Nature.

[16] Luber C.A., Cox J., Lauterbach H., Fancke B., Selbach M., Tschopp J., Akira S., Wiegand M., Hochrein H., O’Keeffe M. (2010). Quantitative proteomics reveals subset-specific viral recognition in dendritic cells. Immunity.

[17] Cox J., Hein M.Y., Luber C.A., Paron I., Nagaraj N., Mann M. (2014). Accurate proteome-wide label-free quantification by delayed normalization and maximal peptide ratio extraction, termed MaxLFQ. Mol. Cell. Proteom..

[18] Wang T., Gong M., Lu Y., Zhao C., Ling L., Chen J., Ju R. (2024). Oxysterol 25-hydroxycholesterol activation of ferritinophagy inhibits the development of squamous intraepithelial lesion of cervix in HPV-positive patients. Cell Death Discov..

[19] Xu D., Zhu X., Ren J., Huang S., Xiao Z., Jiang H., Tan Y. (2022). Quantitative proteomic analysis of cervical cancer based on TMT-labeled quantitative proteomics. J. Proteom..

[20] Aljawad M.F., Faisal A., Alqanbar M.F., Wilmarth P.A., Hassan B.Q. (2023). Tandem mass tag-based quantitative proteomic analysis of cervical cancer. Proteom. Clin. Appl..

[21] Onal C., Guler O.C., Reyhan M., Yapar A.F. (2021). Long-term outcomes of cervical cancer patients with complete metabolic response after definitive chemoradiotherapy. J. Gynecol. Oncol..

[22] Blunsom N.J., Cockcroft S. (2020). CDP-Diacylglycerol Synthases (CDS): Gateway to Phosphatidylinositol and Cardiolipin Synthesis. Front. Cell Dev. Biol..

[23] Pascual G., Majem B., Benitah S.A. (2024). Targeting lipid metabolism in cancer metastasis. Biochim. Biophys. Acta Rev. Cancer.

[24] Chen Y., Xie J., Xie B., Zhong H., Zhang Z., Lin Q., Li E., Yan Z., Cong L., Qin C. (2025). Integrated proteomic and lipidomic analysis revealed potential plasma biomarkers for cervical cancer. J. Pharm. Biomed. Anal..

[25] Sitarz K., Czamara K., Bialecka J., Klimek M., Szostek S., Kaczor A. (2021). Dual Switch in Lipid Metabolism in Cervical Epithelial Cells during Dysplasia Development Observed Using Raman Microscopy and Molecular Methods. Cancers.

[26] Tufail M., Jiang C.H., Li N. (2024). Altered metabolism in cancer: Insights into energy pathways and therapeutic targets. Mol. Cancer.

[27] Kierans S.J., Taylor C.T. (2024). Glycolysis: A multifaceted metabolic pathway and signaling hub. J. Biol. Chem..

[28] Li B., Sui L. (2021). Metabolic reprogramming in cervical cancer and metabolomics perspectives. Nutr. Metab..

[29] Yang J., Cha L., Wang Y., Zhang Q., Tang X., Shao J., Duan Z. (2023). L-Palmitoylcarnitine potentiates plasmin and tPA to inhibit thrombosis. Nat. Prod. Bioprospect..

[30] Liang K. (2023). Mitochondrial CPT1A: Insights into structure, function, and basis for drug development. Front. Pharmacol..

[31] Ma L., Chen C., Zhao C., Li T., Ma L., Jiang J., Duan Z., Si Q., Chuang T.H., Xiang R. (2024). Targeting carnitine palmitoyl transferase 1A (CPT1A) induces ferroptosis and synergizes with immunotherapy in lung cancer. Signal Transduct. Target. Ther..

[32] Liu Z., Liu W., Wang W., Ma Y., Wang Y., Drum D.L., Cai J., Blevins H., Lee E., Shah S. (2023). CPT1A-mediated fatty acid oxidation confers cancer cell resistance to immune-mediated cytolytic killing. Proc. Natl. Acad. Sci. USA.

[33] Liu H., Liu Y., Zhou Y., Chen X., Pan S., Zhou Q., Ji H., Zhu X. (2024). TM7SF2-induced lipid reprogramming promotes cell proliferation and migration via CPT1A/Wnt/beta-Catenin axis in cervical cancer cells. Cell Death Discov..

[34] Yuan L., Jiang H., Jia Y., Liao Y., Shao C., Zhou Y., Li J., Liao Y., Huang H., Pan Y. (2024). Fatty Acid Oxidation Supports Lymph Node Metastasis of Cervical Cancer via Acetyl-CoA-Mediated Stemness. Adv. Sci..

